# *Unc-51*-like kinase (ULK) complex-independent autophagy induced by hypoxia

**DOI:** 10.1007/s13238-018-0584-x

**Published:** 2018-10-29

**Authors:** Yan Feng, Helen H. Kang, Pui-Mun Wong, Minghui Gao, Ping Wang, Xuejun Jiang

**Affiliations:** 1 Shanghai Tenth People’s Hospital of Tongji University, School of Medicine, Tongji University, Shanghai, 200072 China; 2 Cell Biology Program, Memorial Sloan-Kettering Cancer Center, New York, NY 10065 USA; 3 Immunology and Microbial Pathogenesis Program, Weill Cornell Graduate School of Medical Sciences, New York, NY 10065 USA; 4 Human Genetics and Embryology Laboratory; Agency for Science, Technology and Research, Institute of Medical Biology, Singapore, 138632 Singapore; 5 HIT Center for Life Sciences, The School of Life Sciences and Technology, Harbin Institute of Technology, Harbin, 150001 China

**Dear Editor,**

Macroautophagy (referred to as autophagy herein) is an evolutionarily conserved, lysosomal degradation process by which cells rid themselves of aggregated proteins and damaged organelles (He and Klionsky, 2009). This process involves a finely orchestrated molecular pathway comprising a plethora of ATG proteins essential for autophagosome formation. The mammalian Unc-51-like kinase (ULK) complex plays an essential role to initiate canonical autophagy pathway by relaying upstream nutrient and stress signals to downstream autophagy machinery (Mizushima, 2010; Wong et al., 2013). Normally hyperphosphorylated by mechanistic target of rapamycin (mTOR) and thus inhibited in nutrient-rich conditions, the ULK complex can be activated upon nutrient starvation and subsequent mTOR inactivation (Wong et al., 2013). Moreover, nutrient starvation enhances the activity of protein phosphatase 2A (PP2A) to further dephosphorylate ULK1/2 and trigger a more potent and efficient autophagic response (Wong et al., 2015). However, starvation is not the only trigger for autophagic activity. Other stresses can also induce autophagy in many physiologically relevant settings such as cancer. Interestingly, hypoxia, an oxidative stress associated with solid tumor, ischemia, and many other physilogical and pathological conditions (Brahimi-Horn et al., 2007), could induce autophagy as a survival response (Mazure and Pouyssegur, 2010). Nonetheless, the mechanism by which hypoxia induces autophagy is not well defined.

To investigate the molecular mechanism governing the initiation of hypoxia-induced autophagy, we treated wildtype (WT) mouse embryonic fibroblasts (MEFs) with hypoxia (1% O_2_). The results showed that hypoxia induced robust autophagy within 12 h, as a gradual yet robust increase of microtubule-associated protein 1 light chain 3 (MAP1LC3B/LC3, LC3 herein) conversion, decrease of sequestosome 1 (SQSTM1/P62) as well as increase of autophagosome numbers were observed by western blot and GFP-LC3 puncta (Figs. 1A and S1A). Surprisingly, mTORC1 remained mostly active during early hypoxia-induced autophagy (≤12 h 1% O_2_), and significant inactivation of mTORC1 did not occur until 24 h of treatment, whereas serum and amino acid-double starvation for 1 h induced complete mTORC1 inactivation (Fig. 1B). Moreover, robust autophagy was still observed in tuberous sclerosis complex 2 KO (*Tsc2*^−/−^) MEFs (Fig. S1B), in which mTOR is constitutively active and unresponsive to stress stimuli. In conclusion, mTORC1 inactivation was dispensable for early hypoxia-induced autophagy.

**Figure 1:**
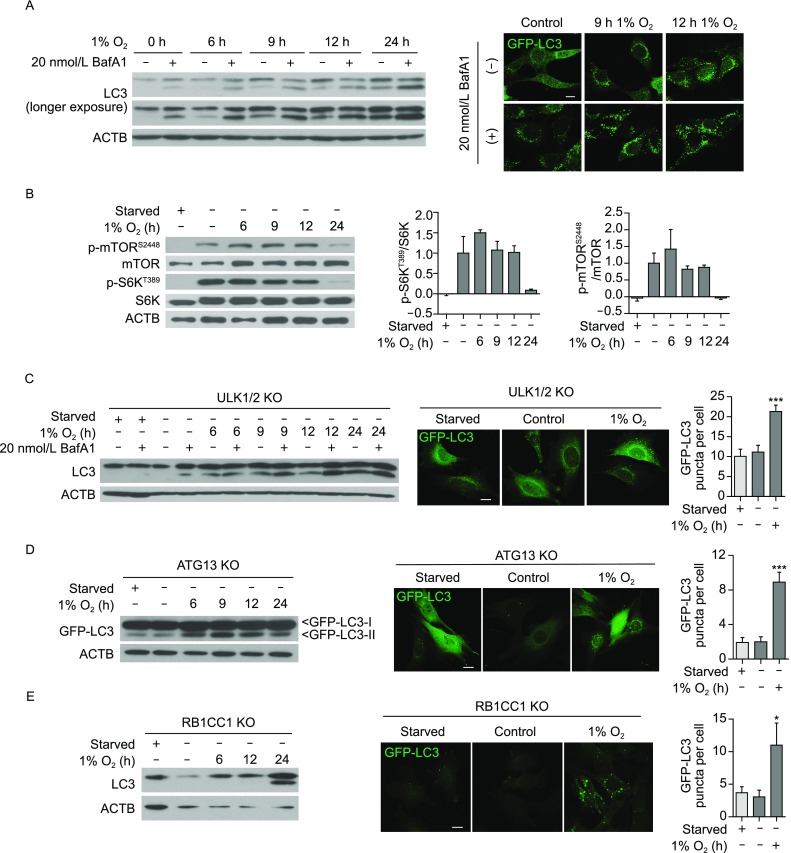
**ULK complex is dispensable for early hypoxia-induced autophagy**. (A, left) Hypoxia (1% O_2_) treatment induced robust autophagy in WT MEFs. WT MEFs were incubated in hypoxia for 6 h, 9 h, 12 h or 24 h, while 0 h (control) cells were incubated in normoxia (21% O_2_) for 24 h. 20 nmol/L bafilomycin A_1_ or vehicle control was added in the last 2 h of treatment. Autophagic turnover was examined by Western blot for LC3B. (A, right) WT MEFs stably expressing GFP-LC3 were used in confocal microscopy to examine autophagosomal puncta formation in response to hypoxia treatment. 20 nmol/L bafilomycin A_1_ or vehicle control was added in the last 2 h of treatment. Scale bar: 10 μm. (B, left) During early hypoxia-induced autophagy in WT MEFs, mTORC1 remained active. Total cell lysates were analysed for mTOR and mTOR substrate S6K phosphorylation status during early hypoxia. As a positive control for mTOR inactivation, 1 h amino acid- and serum-starvation treatment was included. (B, right) Western blot results from two independent experiments were quantified, and show that significant mTORC1 inactivation does not occur until after 24 h 1% O_2_. (C) Hypoxia-induced autophagy occurs in ULK1/2 double KO (ULK1/2 KO) MEFs, as shown by Western blot for LC3-II conversion as well as GFP-LC3 puncta imaging. In contrast to 12 h 1% O_2_ exposure, 1 h amino acid- and serum-starvation did not induce canonical autophagy in these cells. Scale bar: 10 μm. GFP-LC3-positive autophagosomal puncta were counted in at least two independent experiments per cell line. A total of 15 (starved), 16 (untreated) and 27 (hypoxia-treated) cells were analysed. (D and E) Hypoxia-induced autophagy also occurs in ATG13 KO and RB1CC1 KO MEFs, as demonstrated by LC3-II or GFP-LC3-II conversion and GFP-LC3 puncta formation after 24 h 1% O_2_. 1 h starvation was included as a negative control. Scale bar: 10 μm. For ATG13 KO MEFs, 26 (starved), 52 (untreated) and 33 (hypoxia-treated) cells were analysed. For RB1CC1 KO MEFs, 15 (starved), 19 (untreated), 15 (hypoxia-treated) cells were analysed. Error bars indicate standard error of the mean. Results are not statistically significant unless noted otherwise

Since the mTORC1-ULK1/2 axis is responsible for activating canonical autophagy, we next investigated whether the ULK complex was involved in autophagy induction following acute hypoxia. Remarkably, we found that ULK1 remained hyperphosphorylated and inactive during early hypoxia response (Fig. S2A). Consistently, nutrient starvation but not early hypoxia induced colocalization of ULK1 complex and lipidated LC3 on developing autophagosome membranes (Fig. S2B). More importantly, MEFs and human cancer cells that lack major components of the autophagy initiation complex (ULK1/2, ATG13, or FIP200/RB1CC1) still underwent hypoxia-induced autophagy (Figs. S2C–F and 1C–E), demonstrating a ULK complex-independent mechanism for autophagy induction.

However, ULK complex can still play a role in autophagy upon prolonged hypoxia, as long-term hypoxia (≥24 h 1% O_2_) caused a suppression of mTORC1 activity (Fig. 1B). It is well established that mTORC1 suppression can lead to the activation of the ULK complex and in turn, enhanced autophagy activity. Moreover, we observed that transcripts for *Map1lc3b* (coding for LC3B), *Ulk1*, autophagy related 5 (*Atg5*) as well as autophagy related 7 (*Atg7*) were upregulated by prolonged hypoxia (Fig. S3A), which prompted us to test the role of the transcriptional factor hypoxia inducible factor 1 (HIF-1α), the master regulator of hypoxic response (Semenza, 2001), in hypoxia-induced autophagy. Under the conditions used in our study, we found that autophagy-related genes were upregulated upon hypoxia in a HIF-1α-dependent manner, as *Hif1a* knockdown suppressed upregulation of *Map1lc3b* and *Ulk1* (Fig. S3B and S3C). Moreover, *Hif1a* knockdown significantly attenuated hypoxia-induced autophagy (Fig. S3D and S3E). Lastly, the observation that transcriptional inhibitor actinomycin D mitigated hypoxia-induced autophagy (Fig. S3F) further supports the notion that *de novo* transcription is important for long-term hypoxia-induced autophagy. Therefore, we suggest a “two-wave” hypothesis for hypoxia-induced autophagy: early hypoxia induces autophagy in the absence of mTOR regulation or ULK engagement; upon prolonged hypoxia, ULK complex is activated due to inactivation of mTORC1, and multiple autophagy genes are upregulated transcriptionally—all these changes contribute to prolonged hypoxia-induced autophagy. We do not know the exact reason why hypoxia increased *Ulk1* mRNA level but not protein level (Figs. S2A and S3A). One possibility is that the increased transcription is offset by increased degradation of ULK1 protein, considering that long-term hypoxia will engage ULK1-dependent autophagy, and that ULK1-dependent autophagy is associated with ULK1 degradation as reported previously (Liu et al., 2016).

We next examined which molecular players are involved in relaying hypoxic signals to the autophagic machinery. Considering that oxidative stress can lead to an accumulation of mitochondria-derived reactive oxygen species (ROS) (Sena and Chandel, 2012) and ROS can activate adenosine monophosphate-activated protein kinase (AMPK) (Hardie et al., 2012), we tested whether ROS-mediated AMPK activation led to autophagy initiation. Firstly, we confirmed hypoxia-induced activation of AMPK by monitoring an activating phosphorylation site threonine 172 on the catalytic subunit AMPKα and phosphorylation of serine 79 on the canonical AMPK substrate acetyl-CoA carboxylase (ACC) (Fig. 2A). Hypoxia-induced activation of AMPK was also observed in MEFs lacking ULK1/2 or autophagy related 13 (ATG13), notably in the absence of mTOR inactivation (Fig. S4A). Importantly, early hypoxia-induced autophagy was abolished in the AMPKα1/2 KO MEFs (Fig. 2B and 2C). Further, overexpressing a dominant negative K45R mutant form of AMPKα2 in ULK1/2 KO MEFs suppressed AMPK activity and hypoxia-induced autophagy while mTORC1 remained active (Figs. S4B and 2D). Additionally, we found ROS was indeed accumulated in hypoxia-treated WT MEFs (Fig. S4C). Blockage of ROS accumulation by two antioxidants, *N*-acetyl-L-cysteine (NAC) or 6-hydroxy-2,5,7,8-tetramethylchroman-2-carboxylic acid (Trolox), inhibited both hypoxia-induced autophagy and AMPK activation in WT MEFs (Fig. 2E and 2F). However, ROS accumulation was not significantly affected by the absence of AMPKα1/2 following hypoxia treatment (Fig. S4D), further suggesting that ROS acts upstream of AMPK. Together, these results demonstrate that ROS-mediated AMPK activation plays a crucial role in regulating ULK-independent autophagy triggered by acute hypoxia.

**Figure 2:**
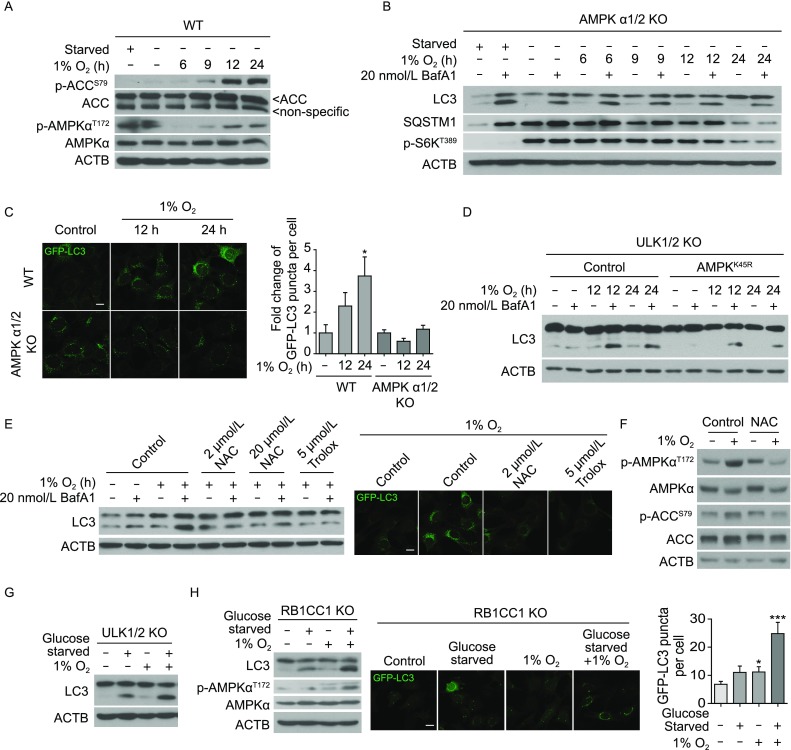
**AMPK regulation is required for early hypoxia-induced, ULK-independent autophagy**. (A) WT MEFs were treated with 0 h, 6 h, 9 h, 12 h or 24 h 1% O_2_, or 1 h amino acid- and serum-starvation. Total cell lysates were analysed by Western blot for phosphorylation statuses of AMPK and its substrate ACC. (B) LC3-II conversion and SQSTM1 degradation were examined using Western blot in AMPK-null (AMPKα1/2 KO) MEFs after 0 h, 6 h, 9 h, 12 h or 24 h hypoxia treatment. 20 nmol/L bafilomycin A_1_ was added in the last 2 h where indicated. 1 h amino acid- and serum-starvation condition was included as a control. (C) AMPKα1/2 KO or WT MEFs stably expressing GFP-LC3 were treated with 0 h (control), 12 h, or 24 h 1% O_2_. Scale bar: 10 μm. Quantification results from two independent experiments were pooled for statistical analyses. In WT MEFs, a total of 28 (untreated), 34 (12 h 1% O_2_) and 36 (24 h 1% O_2_) cells were analysed. In AMPKα1/2 KO MEFs, a total of 25 (untreated), 27 (12 h 1% O_2_) and 28 (24 h 1% O_2_) cells were analysed. 0 h control images were used to normalize puncta count for each group. Error bars indicate standard error of the mean. (D) ULK1/2 KO MEFs overexpressing dominant negative AMPK^K45R^ were treated with 12 h 1% O_2_. Parental ULK1/2 KO MEFs were used as controls. 20 nmol/L bafilomycin A_1_ or vehicle were added in the last 2 h where indicated. (E) Western blot for LC3 conversion and confocal imaging of GFP-LC3 demonstrated that blocking ROS accumulation with antioxidants also blocks hypoxia-induced autophagy. Scale bar: 10 μm. WT MEFs were pre-treated with NAC, Trolox, or vehicle control (DMSO) for 1 h, then exposed to 12 h 1% O_2_. (F) WT MEFs were pre-treated for 1 h with vehicle control (PBS) or 100 µmol/L NAC first, then exposed to 12 h 1% O_2_. These cells were analysed by western blot for AMPKα^T172^ and ACC^S79^ phosphorylation statuses. (G) ULK1/2 KO MEFs were treated with 5 h glucose starvation, 5 h 1% O_2_, or 5 h of both treatments simultaneously. Total cell lysates were examined by Western blot for LC3-II conversion. (H) RB1CC1 KO MEFs were treated with 4 h glucose starvation, 4 h 1% O_2_, or 4 h of both treatments. In these cells, LC3-II conversion as well as AMPK activation was analysed by western blot. RB1CC1 KO MEFs stably expressing GFP-LC3 were exposed to glucose starvation, hypoxia, or both for 4 h, then analysed for autophagosomal puncta formation using confocal microscopy. Scale bar: 10 μm. The number of GFP-LC3-positive puncta per cell were pooled from two independent experiments and plotted. A total of 56 (untreated), 30 (glucose starved), 30 (hypoxia-treated) and 19 (glucose starved and hypoxia-treated) cells were analysed. Error bars indicate standard error of the mean

In the context of glucose starvation, AMPK directly phosphorylates a phosphoinositide-3-kinase, class 3 (PIK3C3) complex subunit, beclin 1, autophagy related (BECN1), at serine 93 to promote autophagy (Kim et al., 2013). Therefore, we sought to determine whether PIK3C3 complex or BECN1^S93^ phosphorylation is involved in AMPK-mediated hypoxia. The PIK3C3 inhibitor SAR405 led to decreased LC3 conversion and autophagy flux upon hypoxia in ULK1/2 KO MEFs, despite of AMPK activation (Fig. S4E and S4F), indicating that PIK3C3 mediates autophagy downstream of AMPK. However, unlike glucose starvation, acute hypoxia (≤12 h 1% O_2_) failed to stimulate BECN1^S93^ phosphorylation in WT or ULK1/2 KO MEFs (Fig. S5A and S5B). Therefore, upon hypoxia, AMPK may phosphorylate BECN1 at a different site, or act on a different downstream component than BECN1. Interestingly, Mec1/ATR, a DNA repair kinase, has been recently identified as a crucial regulator for glucose starvation-induced autophagy in a Snf1/AMPK dependent manner in yeast (Yi et al., 2017). In mammalian cells, although ataxia telangiectasia and Rad3 related protein (ATR) was activated upon acute hypoxia (Fig. S5C), early hypoxia-induced autophagy was not blocked by *Atr* knockdown (Fig. S5D). Taken together, our results suggest that glucose deprivation and hypoxia induce autophagy via distinctive mechanisms, although both engaging AMPK. This conclusion is further confirmed by an additive effect on autophagy activation upon combination of hypoxia and glucose deprivation (an ischemia-like condition) in both ULK1/2 KO and RB1 inducible coiled-coil 1 (RB1CC1) KO cell lines (Fig. 2G and 2H).

Additionally, consistent with the previous report that hypoxia can induce FUN14 domain containing 1 (FUNDC1)-mediated mitophagy (Liu et al., 2012), we found that early hypoxia induced modest mitophagy under the experimental condition used in our study (Fig. S6A and S6B). However, early hypoxia-induced autophagy was not blocked in FUNDC1-eliminated cells (Fig. S6C). Therefore, hypoxia-induced autophagy targets the degradation of both mitochondria (i.e., mitophagy) and other cargos.

In conclusion, we have demonstrated that acute hypoxia can trigger autophagy via a mechanism that is distinctive from that of canonical autophagy. Under this condition, mTORC1 inactivation and subsequent ULK complex activation are not required for autophagy activation; on the other hand, ROS accumulation and AMPK activity play crucial role in acute hypoxia-induced autophagy. Further investigation is needed to define the precise mechanisms of this novel, ULK complex-independent autophagy process. Importantly, this finding also has clear clinical implication. Given its undisputed importance in canonical autophagy induction, ULK has been investigated extensively as a potential therapeutic target. Notably, a novel, highly specific ULK1 kinase and autophagy inhibitor (SBI-0206965) was reported to synergize with nutrient starvation and mTOR inhibition to enhance cancer cell death *in vitro* (Egan et al., 2015). However, it should be unambiguously determined for each individual clinical condition whether autophagy promotes cancer cell survival and whether cancer cell autophagy is ULK complex-dependent, before a future ULK-targeted treatment is applied to patients.

## Electronic supplementary material

Below is the link to the electronic supplementary material.

Supplementary material 1 (DOCX 11701 kb)
