# Supplementary material for: Unc-51-like kinase (ULK) complex-independent autophagy induced by hypoxia
Source: Protein Cell. 2018 Oct 29;10(5):376–81. doi: 10.1007/s13238-018-0584-x (PMC6468041; doi:10.1007/s13238-018-0584-x)
Supplement: Supplementary file 1 — Supplementary material 1 (DOCX 11701 kb) [file 13238_2018_584_MOESM1_ESM.docx]

# Supplemental Material

# Materials & Methods

# *Cell culture and treatments*

Cells were maintained in high-glucose Dulbecco’s modified Eagle’s medium (DMEM) with 10% fetal bovine serum, 1 mM sodium pyruvate, 2 mM L-glutamine, and 10 units/ml penicillin-streptomycin, in a 37ºC/ 5% CO_2_ incubator. Where indicated, cells were subjected to either nutrient starvation (amino acid- and serum-deprivation) or glucose starvation. In such cases, cells were first washed twice with 1x phosphate buffered saline (PBS), then incubated in appropriate starvation media. Fully fed control cells were also subjected to the same washing procedures. For NAC, Trolox, actinomycin D, and SAR405 treatment, cells were first pre-treated for 1 h with either vehicle control or drug. DCFDA (2',7'-dichlorodihydrofluorescein) was added to the last 1 h of culture to measure ROS accumulation. Bafilomycin A_1_ or vehicle was added for the last 2 h of culture to show autophagic turnover. All hypoxic experiments were carried out in a humidified hypoxia workstation (Baker Ruskin, InvivO_2_ 400) at 1% O_2_, 94% N_2_, and 5% CO_2_. Matched normoxic control cells were cultured in a standard tissue culture incubator at atmospheric O_2_ (21%) and 5% CO_2_.

## Knockout and knockdown cell lines

*Ulk1^tm1Thsn^ Ulk2^tm1Thsn^* (ULK1/2 KO), *Prkaa1^-/-^ Prkaa2^-/-^* (AMPKα1/2 KO), and *Prkaa1^+/+^ Prkaa2^+/+^* (WT) MEFs were generous gifts from Dr. Craig Thompson at Memorial Sloan-Kettering Cancer Center in New York, NY, USA. ATG13 KO MEFs (targeting sequence 5’- ACTGTCCAAGTGATTGTCC -3’) and RB1CC1 KO MEFs (targeting sequences 5’- ATGAAAAGATCTACTGAAC -3’ and 5’- ATGATGTGGAATCTCTGGT -3’) were generated using CRISPR-Cas9 system. *Hif1a* was silenced in MEFs using 2 different pLKO-based shRNA sequences, selected for the highest knockdown efficiency: 5’- GTGATGAAAGAATTACCGAAT -3’ and 5’- TGCTCTTTGTGGTTGGATCTA -3’. The target sequences of *Atr* and *Fundc1* were 5’- GATTATTGAATGGGTGAACAA -3’ and 5’- GACTTTATCAAGCAGAACATT-3’ respectively. All the shRNA plasmids were generated by the RNAi core facility at Memorial Sloan-Kettering Cancer Center. For control, a non-targeting sequence was used (5’- C​A​A​C​A​A​G​A​T​G​A​A​G​A​G​C​A​C​C​A​A -3’). WT MEFs were lentivirally transduced with above shRNA vectors as indicated, and stable cell lines were generated using puromycin selection. In U-87 MG glioblastoma cell line, human ULK1 was knocked down using a doxycycline-inducible, pTRIPZ-based shRNA sequence (5’ - CGCCCTTTGCGTTATATTGTAT – 3’). In HT1080 fibrosarcoma cell line, human ATG13 was knocked out using doxycycline-inducible CRISPR-Cas9 system (targeting sequence 5’ – AATGTGCGGAGATGAACCG - 3’). These human cancer cell lines were pre-treated with 500 ng/ml or 1 µg/ml of doxycycline, respectively, for 7 days to ensure efficient knockdown or knockout before assay.

## Antibodies and reagents

The following antibodies were used for western blots shown in this study: Rabbit anti-LC3B (Sigma-Aldrich, L7543), mouse anti-ACTB (Sigma-Aldrich, A1978), rabbit anti-phospho-mTOR^S2448^ (Cell Signalling Technology, 2971), rabbit anti-mTOR (Cell Signalling Technology, 2983), rabbit anti-phospho-S6K^T389^ (Cell Signalling Technology, 9205), rabbit anti-S6K (Cell Signalling Technology, 9202), rabbit anti-SQSTM1 (MBL, PM045), rabbit anti-ULK1 (Sigma-Aldrich, A7481), rabbit anti-ATG13 (Sigma-Aldrich, SAB4200100) mouse anti-GFP (Roche, 11814460001), rabbit anti-HIF-1α (Cell Signalling Technology, 3716), rabbit anti-phospho-ACC^S79^ (Cell Signalling Technology, 11818), rabbit anti-ACC (Cell Signalling Technology, 3676), rabbit anti-phospho-AMPKα^T172^ (Cell Signalling Technology, 2535), rabbit anti-AMPK (Cell Signalling Technology, 5831), rabbit anti-phospho-BECN1^S93^ (Cell Signalling Technology, 14717), and rabbit anti-BECN1 (MBL, PD017), mouse anti-Cytochrome C (BD Biosciences, 556433), rabbit anti-TOM20 (Santa Cruz, sc-11415), rabbit anti-FUNDC1 (EMD Millipore, ABC506), rabbit anti-phospho-ATR^S428^ (Cell Signalling Technology, 2853), mouse anti-ATR (GeneTex, GTX70109), mouse anti-Vinculin (Sigma, V4505), rabbit anti-GAPDH (Cell Signalling Technology, 2118). Rabbit anti-phospho-ULK1^S637^ and rabbit anti-phospho-ULK1^S757^ antibodies were generous gifts from Dr. Xiaodong Wang at University of Texas Southwestern Medical Center in Dallas, TX, USA. The following reagents were used for experimental treatments in this study: Bafilomycin A_1_ (Sigma-Aldrich, B1793), Actinomycin D (Sigma-Aldrich, A9415), NAC (Sigma-Aldrich, A9165), Trolox (Sigma-Aldrich, 238813), DCFDA (Life Technologies, D-399), and SAR405 (ApexBio, A8883).

## Quantitative real time PCR (qRT-PCR)

Following treatment, cell pellets were collected by a low-speed spin and total RNA extracted using Aurum Total RNA Mini Kit (BioRad, 7326820). Equal amounts of total RNA were used to synthesize cDNA with iScript cDNA Synthesis Kit (BioRad, 170-8890). Then, the following primers were used for qRT-PCR: *Map1lc3b* forward (5’- TTATAGAGCGATACAAGGGGGAG -3’), *Map1lc3b* reverse (5’- CGCCGTCTGATTATCTTGATGAG -3'), *Hif1a* forward (5’- ACCTTCATCGGAAACTCCAAAG -3'), *Hif1a* reverse (5’- ACTGTTAGGCTCAGGTGAACT -3'), *Atg5* forward (5’- TGTGCTTCGAGATGTGTGGTT -3'), *Atg5* reverse (5’- GTCAAATAGCTGACTCTTGGCAA -3'), *Atg7* forward (5’- GTTCGCCCCCTTTAATAGTGC -3'), *Atg7* reverse (5’- TGAACTCCAACGTCAAGCGG -3'), *Ulk1* forward (5’- TGGAGGTGGCCGTCAAATG -3'), *Ulk1* reverse (5’- CGCATAGTGTGCAGGTAGTC -3'), *Actb* forward (5’- GTGGCTACAGCTTCACCACC -3'), *Actb* reverse (5’- AGGATGGAGCCACCGATCC -3'). All qRT-PCR was carried out on a BioRad MyiQ instrument.

## Fluorescence microscopy

Cells were plated onto sterile, uncoated glass coverslips in six-well tissue culture plates, between 12 to 24 h before starting treatment. After treatment, coverslips with cells were fixed with 3.7% paraformaldehyde in 20 mM HEPES pH 7.5 for 20 min at room temperature. Following permeabilization with 0.1% Triton X-100, visualization of autophagy related proteins was achieved using immunofluorescence with the following antibodies: Goat anti-WIPI2 (Santa Cruz Biotechnology, sc-83067), mouse anti-ATG16L1 (MBL, M150-3), rabbit anti-ULK1 antibody (Sigma-Aldrich, A7481), rabbit anti-LC3B (MBL, M152-3), Alexa Fluor 594-conjugated goat anti-rabbit antibody (Invitrogen, A11012), Alexa Fluor 488-conjugated goat-anti-mouse antibody (Invitrogen, A11029), and FITC-conjugated donkey-anti-goat antibody (Santa Cruz Biotechnology, sc-2024). Alternatively, MEFs stably expressing GFP-LC3 were generated by retroviral transduction. After PBS washes, coverslips were mounted on glass slides using ProLong Gold mounting media (Life Technologies, P36935). Confocal images were taken at 40x or 60x magnification, using Nikon Eclipse Ti-U microscope with Nikon EZ-C1 image acquisition software. Wide-field images were taken using Nikon TE2000-U microscope and SPOT image acquisition software. Subsequently, raw images were processed using Photoshop software. Images shown are representative images from two or more independent experiments. Quantification of GFP-LC3 or ULK1-positive puncta were performed manually. Confocal image acquisition settings were calibrated to exclude background autofluorescence, based on parental non-fluorescent MEFs. Bright, distinct structures in the cytoplasm were counted as LC3- or ULK1-positive puncta. On average, GFP-LC3-positive puncta showed 14.9 times greater GFP fluorescence than background signal (in contrast to 2.3 times background signal in non-puncta regions within the cell). Average immunofluorescence signal against ULK1 was 2.16 times greater in ULK1-positive puncta compared to that of the background.

##### Flow Cytometry

WT MEFs stably expressing EGFP-Su9 (subunit 9 of the mitochondrial F1Fo-ATPase) (using a PQCXIP vector) were used for flow cytometry analysis. Cells were harvested with trypsin digestion upon hypoxia treatment for indicated time, fixed with the fixation buffer (BioLegend, 420801) for 10 min at room temperature, washed twice and resuspended with PBS buffer. Then the cells were flow cytometrically analysed on LSRIIs (BD Biosciences). Fluorescence intensity of EGFP-Su9 was calculated by FlowJo software and normalized to normoxic control cells (set to 100%).

## Statistical analysis

Data was analysed using GraphPad Prizm 5 software. Unpaired, two-tailed Student’s *t*-test was used for single comparisons. Any *p* value ≤ 0.05 were considered statistically significant. Error bars indicate standard error of the mean. Statistical significance is indicated on graphs as follow: *p* > 0.05; *, *p* ≤ 0.05; **, *p* ≤ 0.01, ***, and *p* ≤ 0.001.
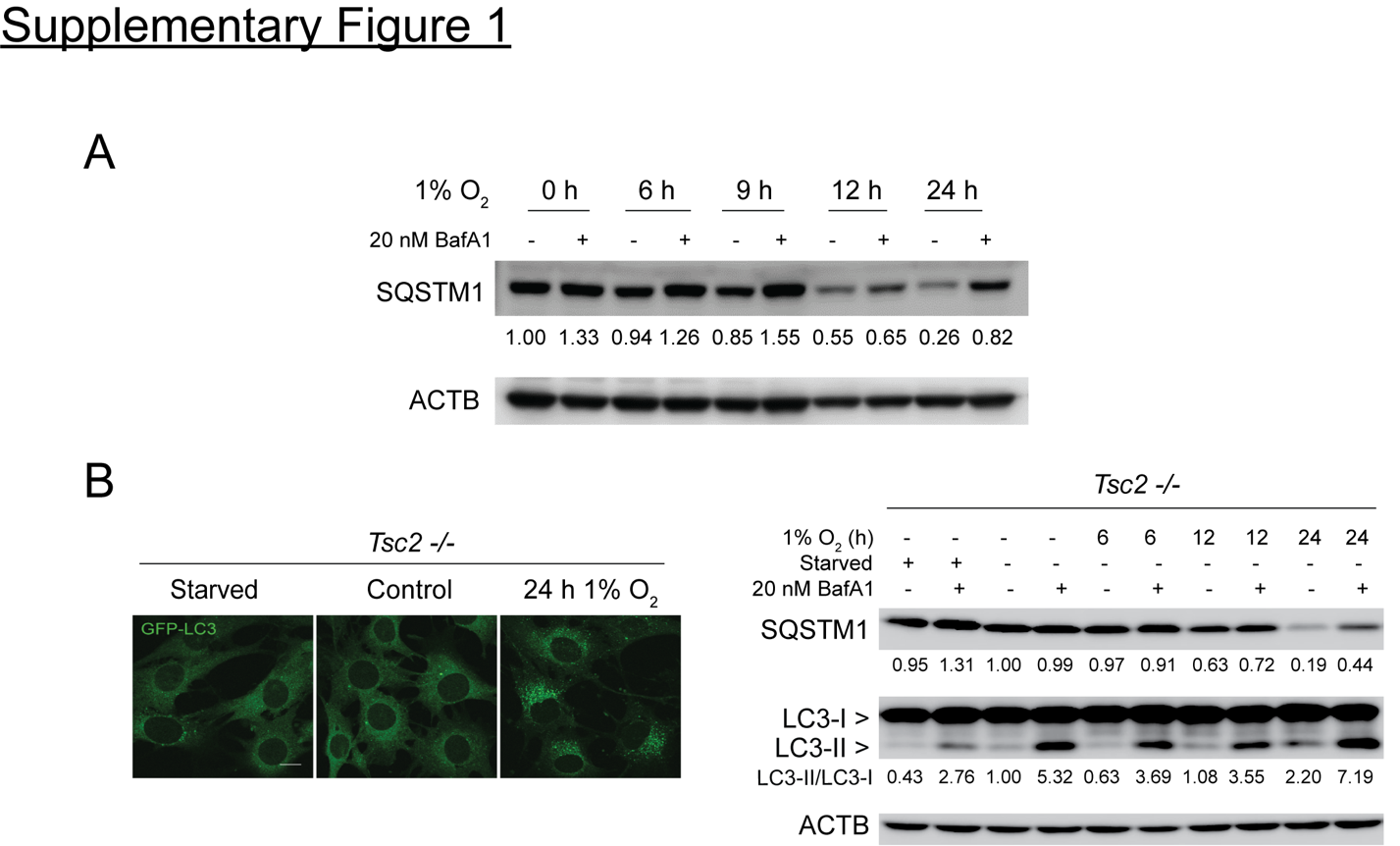


**Supplementary Figure 1. mTORC1 inactivation is dispensable for hypoxia-induced autophagy in MEFs.** (**A)** Hypoxia treatment of WT MEFs resulted in gradual SQSTM1 degradation in a time-dependent manner. WT MEFs were incubated in hypoxia for 0 h, 6 h, 9 h, 12 h, or 24 h (0 h control cells were incubated in normoxia for 24 h). 20 nM bafilomycin A_1_ or vehicle control was added in the last 2 h of treatment. SQSTM1 was quantified by image J and normalized to normoxia condition as indicated. (**B, left**) Hypoxia-induced autophagy was observed in TSC2-null MEFs, in which mTORC1 is dysregulated. Confocal microscopy images showed that GFP-LC3-positive autophagosomes accumulated in TSC-null MEFs after hypoxia treatment. In contrast, 1 h amino acid- and serum- starvation treatment did not increase autophagosome formation. Scale bar: 10μM. (**B, right**) Hypoxia treatment of TSC2-null MEFs resulted in increased LC3-II conversion and SQSTM1 degradation in a time-dependent manner. SQSTM1 and LC3-II/LC3-I ration were quantified by image J and normalized against the normoxic condition. 20 nM bafilomycin A_1_ or vehicle control was added in the last 2 h of treatment to demonstrate autophagy flux. 1 h amino acid- and serum-starvation treatment was included to demonstrate mTORC1 dysregulation in these cells.


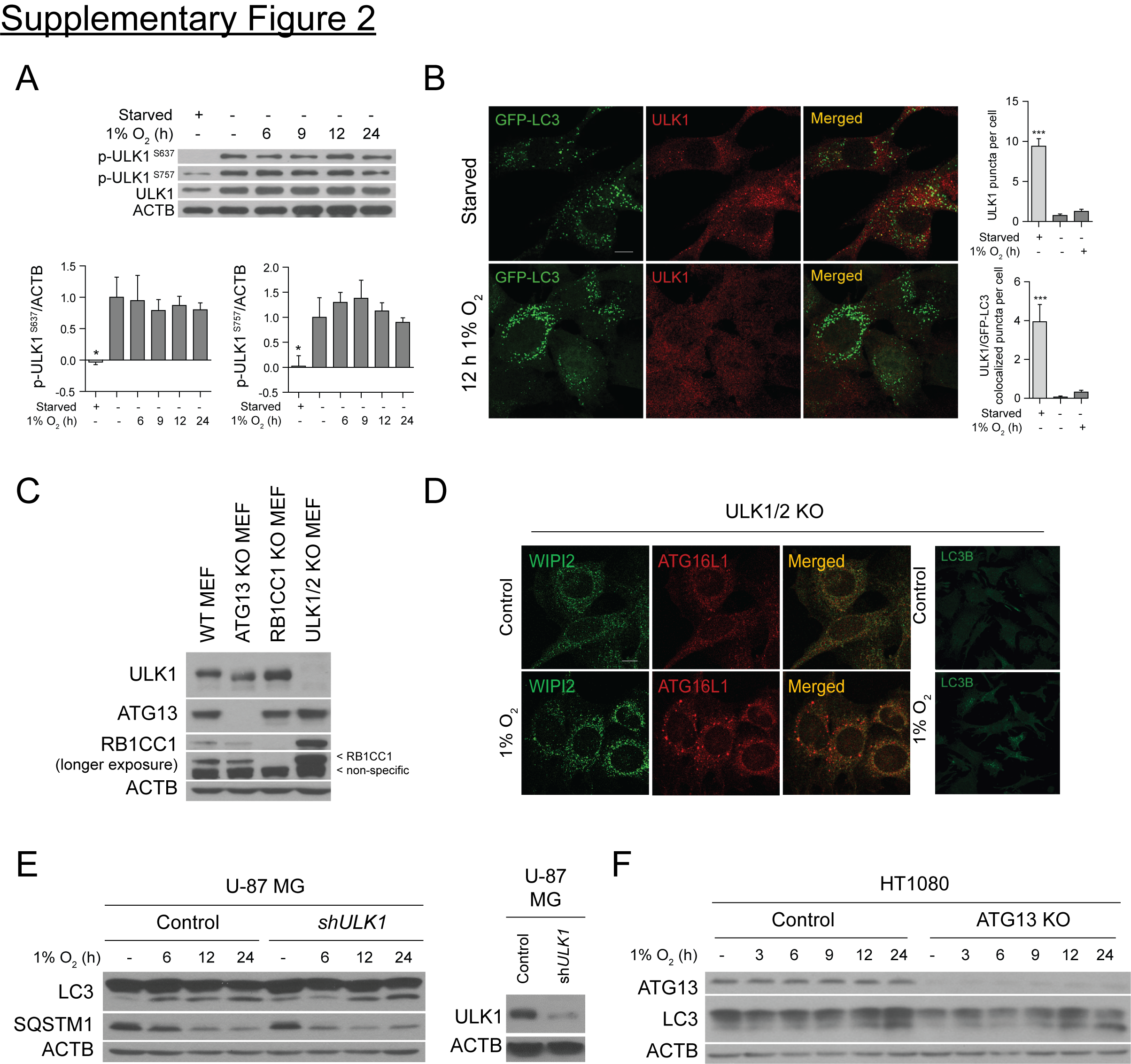


**Supplementary figure 2. ULK complex is not required for hypoxia-induced autophagy.** (**A, top**) During early hypoxia-induced autophagy, ULK1 remained phosphorylated at two known mTORC1-regulated serine residues, 637 and 757. In contrast, 1 h starvation leads to a clear dephosphorylation of ULK1 at these residues. (**A, bottom**) Western blot results from three independent experiments were quantified, to show that ULK1 remained phosphorylated during early hypoxia. (**B**) Confocal microscopy images showed ULK1 immunofluorescence in WT MEFs stably expressing GFP-LC3. ULK1 immunofluorescence showed that ULK1 did not localize to GFP-LC3-positive autophagosomes after 12 h of hypoxia treatment. In contrast, in line with the evidence from western blotting, starvation caused a clear colocalization of ULK1 and GFP-LC3 puncta. ULK1-positive puncta as well as ULK1/GFP-LC3-colocalized puncta were counted from two independent experiments. For ULK1 puncta quantification, a total of 35 (starved), 42 (untreated), and 40 (hypoxia-treated) cells were analysed. ULK1/GFP-LC3 colocalization was analysed in a total of 19 (starved), 16 (untreated), and 25 (hypoxia-treated) cells. Scale bar: 5μM. (**C**) KO status of MEFs deficient in ULK complex components were determined by western blotting. For RB1CC1, RB1CC1-specific and non-specific background bands are indicated. (**D**) Following 24 h hypoxia (1% O_2_) or control normoxic treatment, endogenous autophagy proteins WIPI2, ATG16L1, and LC3 localized to punctate autophagosomal structures upon hypoxia detected by immunofluorescence in ULK1/2 KO MEFs. Scale bar: 5μM. (**E**) Human glioblastoma cells (U-87 MG) were transduced with a doxycycline-inducible shRNA against ULK1. Stable cell lines that had been cultured with and without 500 ng/ml doxycycline for 7 days (sh*ULK1* and control, respectively) were then subjected to 0 h, 6 h, 12 h, or 24 h 1% O_2_. Efficient ULK1 protein knockdown was confirmed in normoxic cells. (**F**) A human fibrosarcoma cell line (HT1080) was transduced with a doxycycline-inducible CRISPR-Cas9 system targeting ATG13. HT1080 cells that had been pre-treated for 7 days with 1 µg/ml doxycycline were subsequently exposed to 0 h, 3 h, 6 h, 9 h, 12 h, or 24 h 1% O_2_, then analysed by western blot.


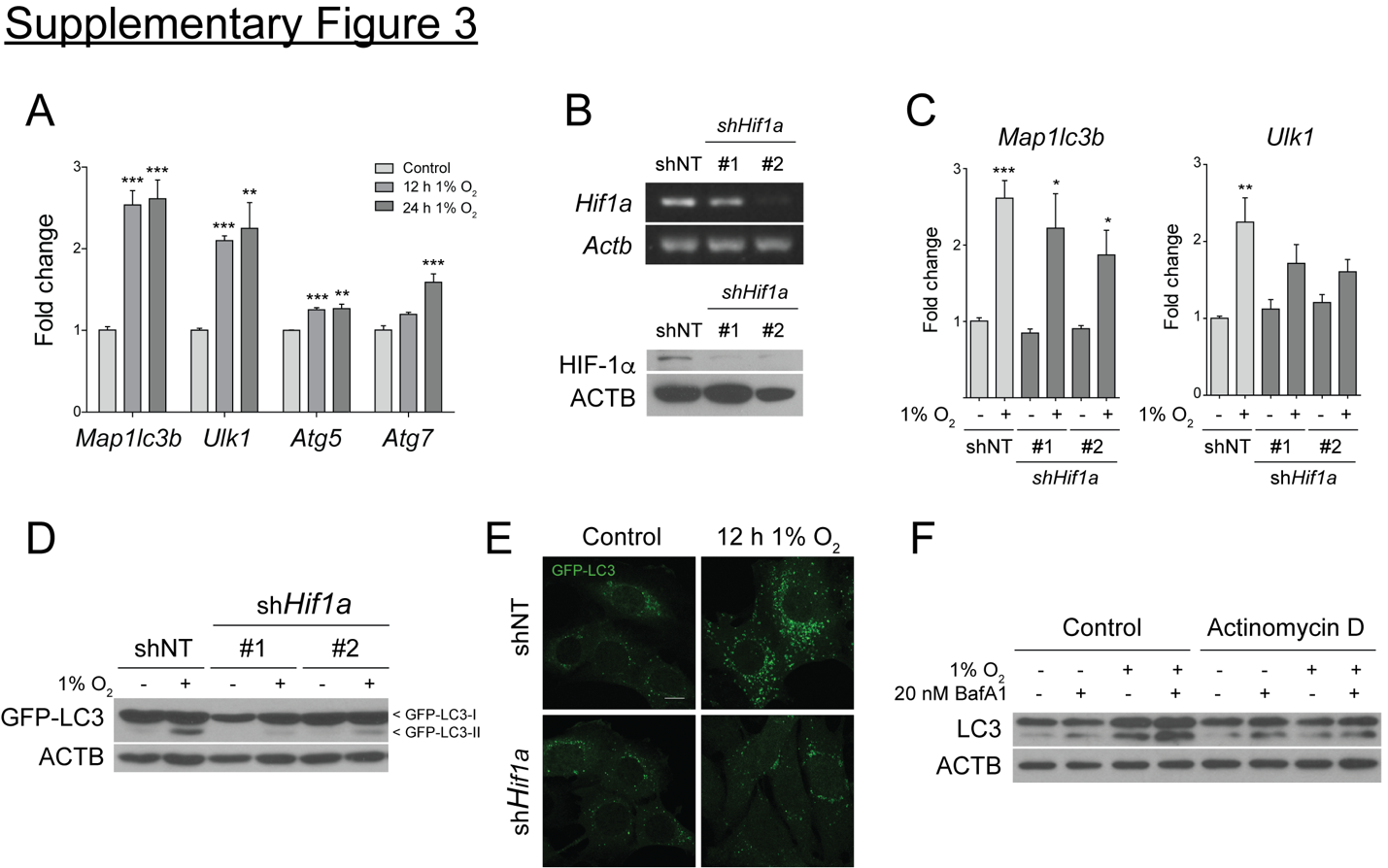


**Supplementary figure 3*.*** **Hypoxia induces transcriptional upregulation of autophagy genes in a** **HIF-1α-dependent manner.** (**A**) qRT-PCR revealed that hypoxia treatment (12 h and 24 h 1% O_2_) in WT MEFs induced upregulation of autophagy genes *Map1c3b*, *Ulk1*, *Atg5*, and *Atg7*. Control (normoxic) samples were used to normalize each transcript level. Data from two independent experiments were pooled and statistically analysed using Student’s *t*-test. Error bars indicate standard error of the mean. (**B**) *Hif1a* was efficiently silenced by shRNA in MEFs. *Hif1a* was knocked down in WT MEFs using 2 different shRNA sequences (sh*Hif1a* #1 and #2). A non-targeting sequence (shNT) was used as control. RT-PCR (top) and western blotting (bottom) showed efficient knockdown of *Hif1a* transcript and HIF-1α protein, respectively. (**C**) When WT MEFs stably transfected with *Hif1a* knockdown constructs were treated with 24 h 1% O_2_, we observed a notable decrease in hypoxia-induced autophagy gene transcript upregulation. For both *Map1c3b* and *Ulk1*, mRNA levels were normalized using the normoxic shNT control. Data from three independent experiments were pooled and statistically analysed using Student’s *t*-test. Error bars indicate standard error of the mean. (**D**) *Hif1a* knockdown in WT MEFs led to decreased GFP-LC3-II conversion after 24 h 1% O_2_. (**E**) Confocal imaging showed that GFP-LC3-positive puncta formation was blocked in cells stably expressing sh*Hif1a*#2. Scale bar: 5μM. (**F**) *De novo* transcription and translation was required for hypoxia-induced autophagy. Cells were pre-treated with 0.5 µg/ml actinomycin D or vehicle control for 1 h, then subjected to 12 h 1% O_2_ hypoxia or normoxia, then analysed for endogenous LC3-II conversion. 20 nM bafilomycin A_1_ was added in the last 2 h where indicated.

**
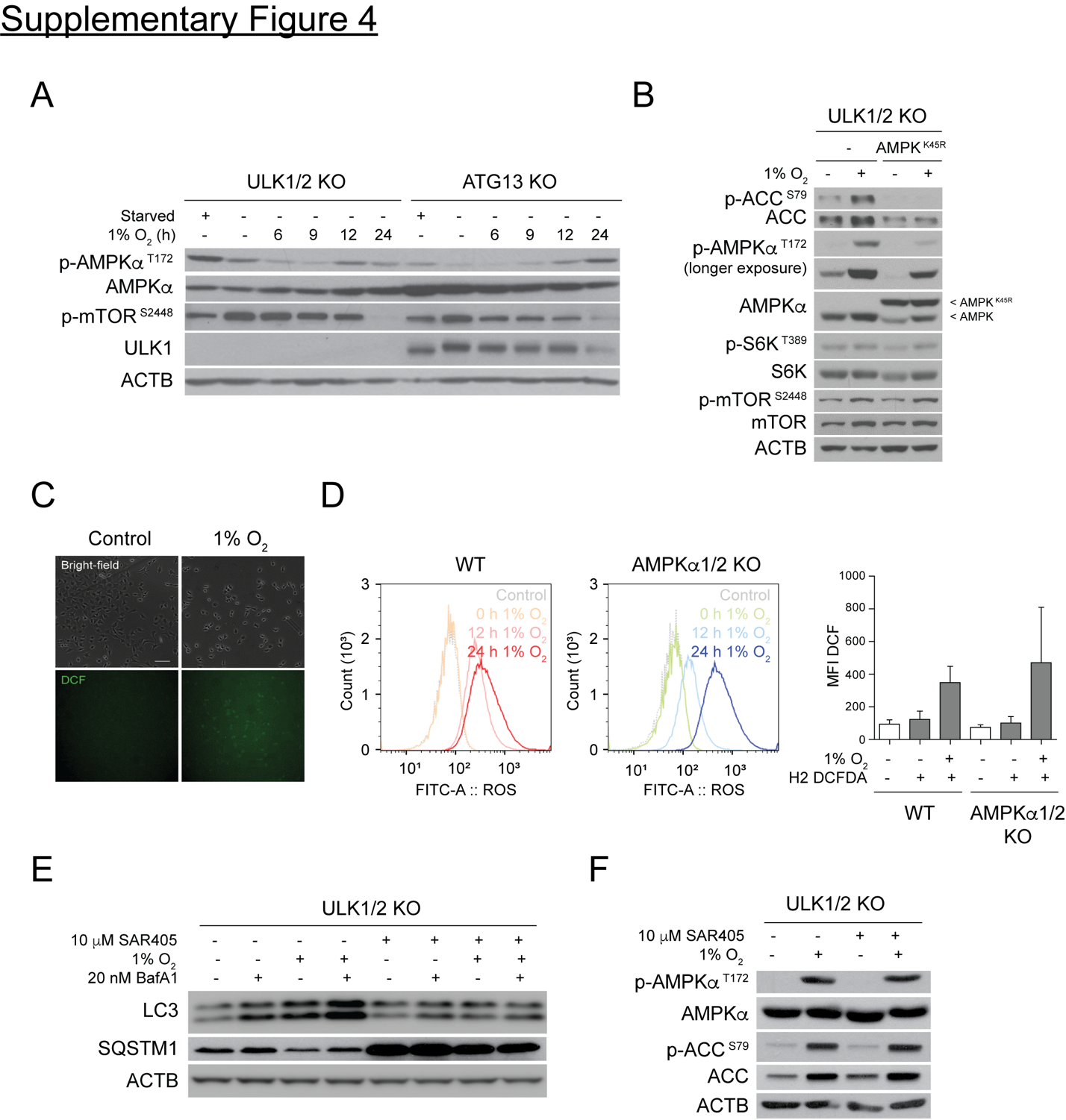
**

**Supplementary figure 4. Both ROS accumulation and the VPS34 complex play a role in early hypoxia-induced, ULK-independent autophagy by activating AMPK.** (**A**)The kinetics of hypoxia-induced AMPK activation and mTOR inactivation in MEFs remained comparable in the absence of ULK1/2 or ATG13. Phosphorylation statuses of AMPK and mTOR were analysed by western blot in ULK1/2 KO MEFs or ATG13 KO MEFs upon 0 h, 6 h, 9 h, 12 h, or 24 h 1% O_2_. 1 h amino acid- and serum-starvation condition was used as control. (**B**) Overexpression of dominant negative AMPK in ULK1/2 KO MEFs abolished AMPK activity without affecting mTORC1. Cells were treated with 12 h 1% O_2_, and analysed by western blot. Parental ULK1/2 KO MEFs were used as control. Endogenous WT AMPK and exogenous AMPK^K45R^ were indicated accordingly. (**C**) Fluorescence microscopy imaging showed DCF signal in WT MEFs after 24 h incubation in hypoxia, indicating accumulation of ROS in these cells. Scale bar: 200μM. **(D)** ROS accumulated similarly in WT and AMPKα1/2 null MEFs. Following hypoxia exposure in WT and AMPKα1/2 KO MEFs, total cellular ROS was measured using H_2_DCFDA by flow cytometry. Representative FACS plot showed comparable time-dependent ROS accumulation in WT and KO MEFs. Results at 12 h 1% O_2_ from two independent experiments were quantified on the graph. (**E-F**) The VPS34 complex was required for hypoxia-induced, ULK-independent autophagy. ULK1/2 KO MEFs were pre-treated with vehicle control (DMSO) or 10 µM of the VPS34 inhibitor, SAR405 for 1 h first. After 24 h hypoxia or normoxia treatment, total cell lysates were harvested and analysed by western blot for LC3-II conversion and SQSTM1 degradation (**E**) as well as AMPK activation status (**F**). 20 nM bafilomycin A_1_ was added in the last 2 h where indicated.


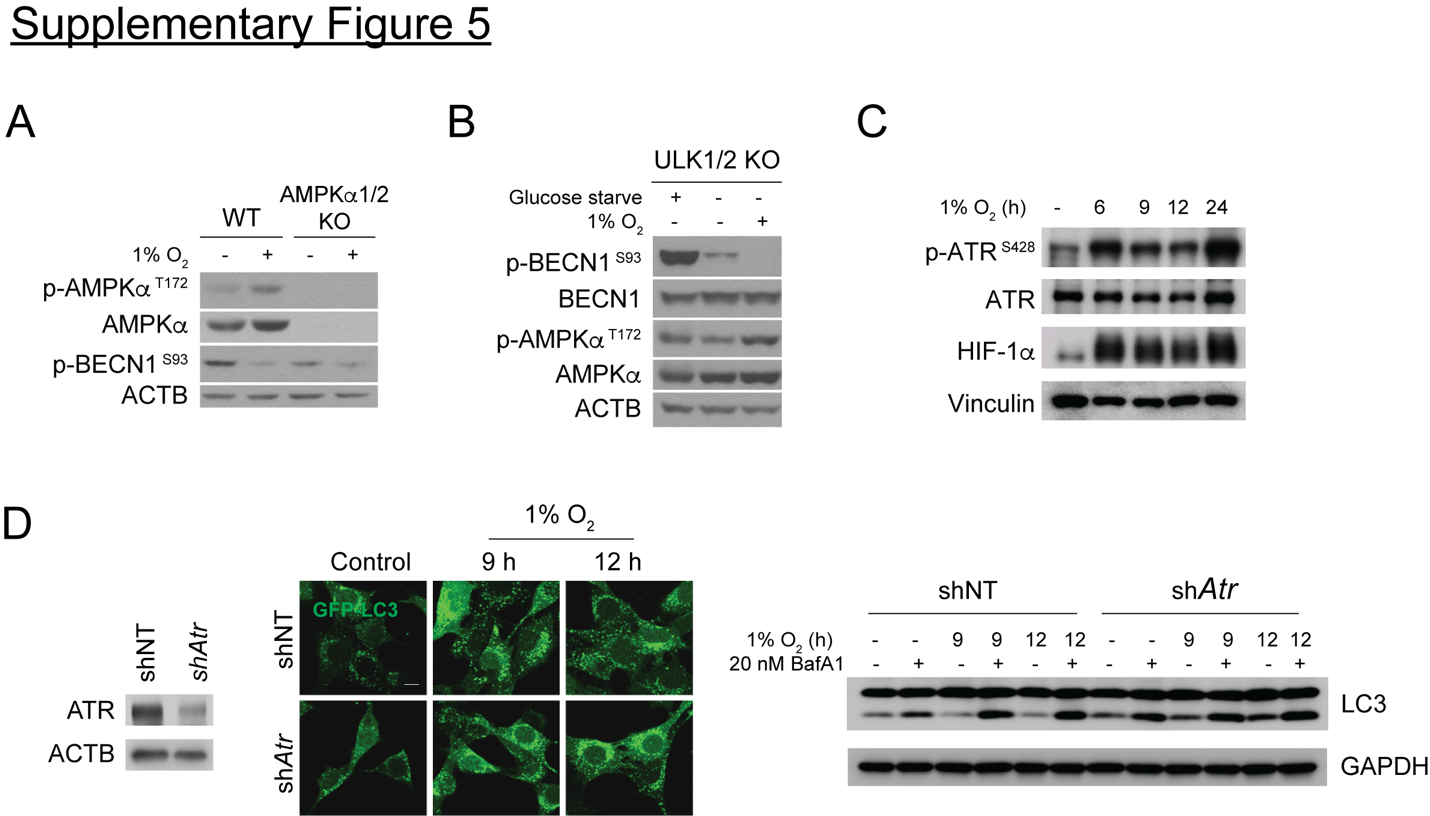


**Supplementary figure 5. Neither BECN1^S93^ phosphorylation nor ATR is involved in AMPK-mediated autophagy during early hypoxia.** (**A**) WT and AMPKα1/2 KO MEFs were exposed to 12 h 1% O_2_ and analysed by western blot. While hypoxia induced AMPKα^T172^ phosphorylation in the WT MEFs, concurrent BECN1^S93^ phosphorylation was not observed. (**B**) ULK1/2 KO MEFs were treated with 12 h 1% O_2_. 6 h glucose starvation was used as a positive control for BECN1^S93^ phosphorylation. (**C**) ATR was activated by hypoxia in WT MEFs as evaluated by the increased level of ATR^S428^ phosphorylation. Vinculin was used as loading control. (**D**) Left panel, ATR was efficiently silenced by shRNA in MEFs. Middle panel, confocal imaging showed that GFP-LC3 puncta formation was not blocked in cells stably expressing sh*Atr*. 20 nM bafilomycin A_1_ was added in the last 2 h of treatment. Scale bar: 10μM. Right panel, *Atr* knockdown in WT MEFs did not block early hypoxia-induced autophagy. LC3-II conversion was examined using western blotting after 0 h, 9 h or12 h hypoxia treatment. 20 nM bafilomycin A_1_ was added in the last 2 h of treatment where indicated.


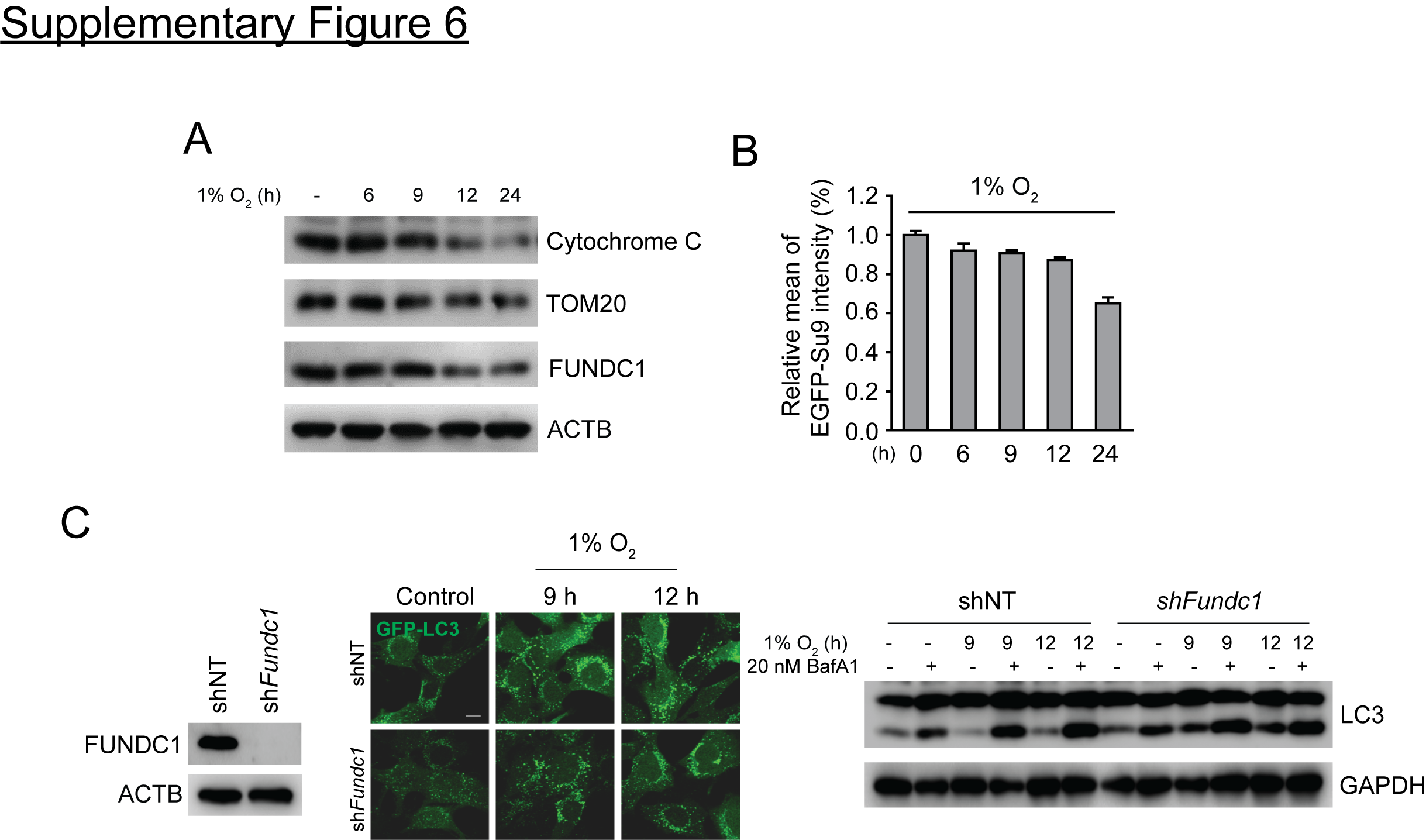


**Supplementary figure 6. FUNDC1 was not required for early hypoxia-induced autophagy.** (**A**) Mitophagy was mildly induced upon hypoxia in WT MEFs. Total cell lysates were analysed for the levels of mitochondria proteins Cytochrome C, TOM20 (translocase of outer membrane) and FUNDC1 during hypoxia treatment at indicated time points. (**B**) WT MEFs stably expressing EGFP-Su9 were used for FACS analysis to detect mitochondria status upon hypoxia at indicated time points. (**C**) Left panel, FUNDC1 was efficiently silenced by shRNA in MEFs. Middle panel, confocal imaging showed that GFP-LC3 puncta formation was not blocked in cells stably expressing sh*Fundc1*. 20 nM bafilomycin A_1_ was added in the last 2 h of treatment. Scale bar: 10μM. Right panel, *Fundc1* knockdown in WT MEFs did not block early hypoxia-induced LC3-II conversion at indicated time points.
